# Technological Advancements in Monoclonal Antibodies

**DOI:** 10.1155/2021/6663708

**Published:** 2021-02-10

**Authors:** José F. Santos-Neto, Fabricia O. Oliveira, Katharine V. S. Hodel, Larissa M. S. Fonseca, Roberto Badaró, Bruna A. S. Machado

**Affiliations:** ^1^University Center SENAI/CIMATEC, National Service of Industrial Learning–SENAI, Salvador, Bahia, Brazil; ^2^SENAI Institute of Innovation (ISI) in Advanced Health Systems (CIMATEC ISI SAS), University Center SENAI/CIMATEC, National Service of Industrial Learning–SENAI, Salvador, Bahia, Brazil

## Abstract

Biopharmaceuticals are innovative solutions that have revolutionized the treatment of important chronic diseases and malignancies. The approval of biosimilar products has become a complex and balanced process, and there are versions of drugs with established biosimilarity that can offer a more accessible treatment option to patients. The objective of this work was to identify the advancement of these technologies by means of patent and article analysis based on technological and scientific prospection. In patent document recovery, Derwent Innovation Index (DWPI) and PatentInspiration databases were used. The research was based on the search of the selected terms in the title, summary, and claims of the documents through a search strategy containing IPC code and keywords. In articles recovery, the Web of Science tool was used in the search of scientific publications dated from the last 5 years. The search resulted in a total of 2295 individual patent documents and 467 families using DWPI database, 769 individual patents and 205 families using PatentInspiration, and 2602 articles using Web of Science database. Additionally, this work describes the number of organizations that contribute to this area, where they are, how much development they have undergone, and the inventors/authors involved. Based on the number of publications registered, there is an important prominence for scientific research in mAbs. In terms of innovation, it is expected that several therapeutic drugs are already under regulatory review, which will allow drugs to be approved over the next few years and will thus generate a continuous flow of new products based on immunotherapies, mAbs, and biosimilar drugs. These drugs have become essential weapons for the treatment of significant diseases, and the increasing trend in the number of related scientific and technological publications contributes to making these therapies available to the greatest number of people.

## 1. Introduction

Biological products (biological or biopharmaceuticals) are compounds formulated from living systems through the application of modern biotechnological methodologies [1]. Their production became possible due to the evolution of molecular biology through the so-called recombinant DNA technology, which uses a variety of expression systems (e.g., bacteria, yeast, and mammalian cells). Over the last decades, progress in this area have allowed the modification and transfection of genes encoding activated biological proteins from one organism to another, with the aim of obtaining highly efficient effectiveness of their products [2].

Officially, biologicals were introduced to the market as “biological drugs” in the early 1980s. In 1982, recombinant human insulin became the first biotechnological therapy to obtain approval from the Food and Drug Administration (FDA) and reach the market for the treatment of diabetic people [3], even though the first two biologicals, insulin (nonhuman) and human growth hormone (HGH), had already been commercialized in the United States market for a long time but were not labeled as biological [4].

Biologicals are generally composed by peptides, proteins, or nucleic acids [5]. Moreover, cell-based systems are also considered biopharmaceuticals or biologicals for application in cell therapy [6]. Normally formed by large and more complex molecules, biologicals differ from drugs formed by small molecules of known chemical structure, as is the case with synthetic drugs [7]. Synthetic drugs are usually more pure and better characterized in terms of current analytical technology, whereas biologicals have a complex mechanism of action, which can be affected by the cellular system in which they are produced, by the fermentation medium or by operational conditions [8].

Despite these and other differences inherent to the biological production and performance process, biologicals are an innovative solution that has revolutionized the treatment of important chronic diseases (e.g., cancer, immune-mediated inflammatory conditions, diabetes mellitus, and infertility) [9–12]; this allows them to be considered as keys for the treatment of other diseases in the future [13], especially for use in targeted therapies against cancer, with considerable growth in the development of new molecules [14]. However, the clinical benefits of biological therapy are negatively balanced with the challenges related to the accessibility of these drugs [15].

The investigation of biologicals directly influences their accessibility due to the high investment costs for their production. For this reason, the approval of biosimilar products is becoming a declared and balanced process [4]. Biosimilars are concepts that may vary according to the regulatory agency. According to the FDA, these products are defined as highly similar drugs with no clinically significant differences from the already approved reference product from which they are derived [16]. Also called follow-on biologics (FOB) or subsequent entry biologics (SEBs), biosimilars are biopharmaceuticals that are produced and sold on the market after the expiration of patents for innovative biological products [17], when technology comes into the public domain and the originating company loses production exclusivity.

Like generic drugs, biosimilars are versions of drugs that can offer a more accessible treatment option to patients once their production is generally less expensive. This may be related to a shortened clinical trial program and possibly to the application of more advanced and efficient technologies during the downstream and upstream processes [18, 19]. Although the cost savings were not as dramatic as expected after the approval of the first biosimilar in the USA (United States of America) [20], the estimated future reduction in spending due to the use of biosimilars is substantial [21, 22]. Therefore, the production segment of these biopharmaceuticals undoubtedly has a major impact on innovative companies, paving the way for pharmaceutical companies in the industry to prosper.

As stated, the main factor for the capture of biosimilars is the reduction of costs in relation to the biological products of origin. This evidence, based on biosimilar value propositions, has recently been well described [23]. In addition, other authors argue that there are other benefits directly linked to cost reduction: the improvement of the cost-benefit ratio of biological therapy [24–28], the treatment access when the disease is still in its early stages, and the patient access to innovative treatments at low cost [29, 30]. Moreover, cost savings can still allow a division between the interested parties, through an “earnings sharing agreement,” with the objective of promoting the capture of biosimilars [15], and can also be reinvested in hiring more healthcare professionals or to improve health resources in a hospital with limited capacity [31, 32] and stimulating innovation by increasing the competitiveness between nonpatented and biosimilar biologicals, which can reflect the production of new formulations, administration routes, and adherence approaches [33].

Regarding production, it is not possible for manufacturing of identical biologicals and biosimilars, even if the manufacture uses the same technology or expression system [34]. However, although their development is complex due to the complexity of biologicals, which is directly related to the large size of the molecules and the complicated process of obtaining them, significant differences between biosimilars and reference biologicals are not expected in relation to quality, safety, purity, and efficacy, since these biosimilars also undergo consistent evaluation by regulatory agencies before being licensed in order for their production and commercialization to occur [19].

According to the World Health Organization (WHO), for a biosimilar candidate to be produced, it is necessary to meet the criteria of biosimilarity in relation to his biological reference. Otherwise, it cannot be considered as biosimilar, even if its production and commercialization are authorized in countries with less restrictive legislative criteria. [2]. Although the WHO is not a regulatory authority, its scope includes the unique role of supporting national agencies in all its member states. More specifically, one of its main functions is “to establish norms and standards to promote and monitor its implementation.” This function has been directly linked to the definition and promotion of global standards for biological products. At the beginning of the 20th century, it was when the standardization of biologicals production was recognized. This standardization was created from the Biological Standardization Commission, established by the League of Nations [35–37]. The Experts Committee on Biological Standardization (ECBS) exists since 1947, and since then, it is responsible for the WHO standardization of biological products.

The regulation about the necessary requirements for the approval of biosimilars in the market was created in several countries. In the case of Brazil, ANVISA (National Health Surveillance Agency) is the entity responsible for this regulation in the country. Although not completely aligned with international trends, the Collegiate Board Resolution 55/2010 (RDC 55/2010, in Portuguese) is the guideline that guides as to the requirements that must be fulfilled for new biological or follow-on products to be approved. This includes not only monoclonal antibodies but also vaccines, blood derivatives, and some living organisms, among others [38].

Monoclonal antibodies (mAbs) are defined as glycoproteins capable of binding an antigen to a specific epitope [39], and in recent years, it has been increasingly reaching maturity in therapy, with several molecules approved by regulatory agencies [40]. Initially, the immunoglobulins were produced from hybridomas, using mammalian cells by the recombinant DNA technology. However, despite being considered the expression system that allows a better production of mAbs in large scale because of advantages such as greater biochemical similarity, greater stability, and higher levels of expression, the use of mammalian cells implies in a longer cycle of production, which generate higher costs [41, 42]. Another system widely chosen for mAbs production is the bacteria *Escherichia coli* because it usually consists of a faster and therefore less costly process, being widely used in academic research and by pharmaceutical industries [41]. mAbs are IgG-type immunoglobulins Y-structured with two identical light chains and two heavy chains, also identical, which connect through disulphide bonds. Both chains have a constant and a variable region that is responsible for conferring specificity to the antibody. The variable domains, Fab or binder fragment of antigen, form the region of binding to the antigen, while the Fc domain (crystallized fragment) is the one that interacts with receptors present on the cell surface and the one that contains a complementarity determining region (CDR), through which the highly specific and functional binding between the antigen epitope and the mAb occurs [43]. A key point that directly influences the functionality of a mAb is the understanding of its pharmacokinetics, which involves not only factors related to the target antigen but also unrelated factors that can influence its distribution during treatment. Another key point is the immunogenicity of the antibody, that is, why the need to perform preclinical safety evaluations that include influencing factors (e.g., study population, route/s of administration, selection of starting dose, and study design) [44]. Since the first approval, research involving the development of mAbs has grown steadily. Currently, more than 500 antibodies are in early stages of research, while more than 50 mAbs are in the final stages of clinical development, most of which are directed at combating cancer and autoimmune or inflammatory diseases such as melanoma, lupus, and rheumatoid arthritis. Regarding the variety of existing mAbs and the amount of diseases that can be treated, infliximab and rituximab biosimilars are among those that have shown great clinical success [44].

In terms of innovation, companies in the biopharmaceutical field have been showing greater attention to the production and marketing of biosimilar drugs [45]. Since 2005, the European Medicines Agency (EMA) has approved more than 40 biosimilar drugs [12]. In 2012, 26 biosimilars were already in advanced stages and in 2015, another 47 in clinical trials [17]. In the past 3 years, 12 biosimilars have been approved by the FDA [46].

On April 27, 2015, the first biosimilar was approved in Brazil by ANVISA. The first monoclonal antibody, Remsima, is produced by the biopharmaceutical company Celltrion, South Korea, which is marketed and distributed in Brazil by Hospira US [47]. The biosimilar infliximab is based on the biological reference drug Remicade, produced by the American company Janssen Biotech, Inc., and distributed in Brazil by the same company [48]. Among biological and protein-based biosimilars, mAbs comprise a functionally complex structural class. Its productions were based on in vitro display techniques, such as bacteria, phages, yeast, bacteria, ribosomes or mRNA display [49], hybridomas [50], or heterologous expression [51].

This and other information about the status of mAbs technologies can be found using various methods. One of them is the use of prospective methods for collecting information. Among the prospective methods, the use of patents has been widespread in recent years. This type of prospecting is widely used for scientific and technological mapping, since the use of sources of information from patents integrate the scope of technological management and innovation activities, constituting themselves as competitive tools used in the evaluation of technological opportunities [52, 53]. Furthermore, they can help in the identification of actors involved in the area of mAbs production and can contribute to the decision making of public institutions and private companies. The same possibilities are found in the mapping conducted through scientific publications. Furthermore, through the analysis of scientific publications, it is possible to analyze the state of the art in relation to this technology, besides anticipating possible trends and acting in the identification of new research in development [54].

It is known that patents are sources for obtaining technological information and companies use them as a strategy for technological management and innovation [55], since the content available in patent documents can direct a mapping of a particular technology, allowing the collection of important information about technological developments, inventors, market trends, and others, as previously mentioned [56–58]. To file a patent means to protect an invention with the right to exclude that other people can develop or make use of the same technology for a certain period of time [55]. Besides the important commercial aspect associated with the protection of inventions by means of patents, it is possible to state that a lot of protected information is not made available in any other source of information, where about 80% of the technological content contained in the form of patents is only found in this type of document [59].

Regarding the commercial aspect related to patents, the possibility of mapping sectors and technologies based on the information contained in these documents allows us to highlight not only the competitiveness of the technology but also how predominant it can be in the commercial market. Among the other purposes, the decision making, the identification of new markets, the forecast of other products, and business opportunities are among the objectives for the realization of a search for patents [60]. All of them aimed at maximizing the contribution to the sector of development and technology, supporting technological management [60] and minimizing the distance between companies, universities, and research institutes, collaborating for possible partnerships in the development of new technologies [58]. The patent indicators can be found in a search for patents through a database available in the market, which can be paid or free of charge, but basically allow the gathering of information and tools for the treatment of data, differentiating between them in relation to the quality of information obtained. Therefore, the choice of the database to be used in the research is fundamental to achieve the desired information [61].

Bringing already a little of the general information found in patent documents, most patents involving mAb technologies address not only the treatment but also the prevention of cancer and autoimmune illnesses [62, 63], as publication number US 2012/0295275 A1, 2012, which deals with the use of mAbs in the identification of cancer cells that express the BFA4 protein. The technology refers to the use of antibodies to identify cells that express the BFA4 protein, as well as for the diagnosis, prevention, and/or treatment of one or more diseases related with the existence of such cells, like ovarian, renal and lung cancer, pancreatic and biliary cancer, B and T cell lymphoma, colon carcinoma, and hepatocarcinoma. [64]. Patent number PI 0608281-5 B1, 2020, refers to antibodies and their fragments that neutralize the increased and/or undesirable activity of GM-CSF (granulocyte-macrophage colony-stimulating factor), a cytokine that belongs to a group of glycoproteins that regulate the proliferation and differentiation of hematopoietic cells, more specifically granulocytes and macrophages-granulocyte colonies, through pharmaceutical compositions comprising antibodies and its fragments that neutralize the activity of this factor. The purpose of the invention is the production of drugs that can be used to treat various inflammatory conditions [65]. In the publication WO 2017/046658 Al, 2017, there is a description of a methodology for the construction of antibody-drug conjugates, based on the ability to target mAbs to bring highly toxic drugs, more specifically amanita toxins, to tumor cells. The therapeutic use of its derivatives can be used in the targeted treatment of cancer, autoimmune disorders, and infectious diseases [66]. Like this patent, several other publications involve the construction of antibody-drug conjugates. Currently, more than 50 drugs of this type are already in the clinical trial phase. Authors emphasize that this technology is considered a potential innovation challenge [67].

In general, targeted therapies using monoclonal antibodies have significantly impacted the treatment of major diseases, and with the advent of biosimilars, accessibility to these products has become possible. Therefore, the objective of this work was to identify the advancement of these technologies by means of patent and article analysis based on technological mapping.

## 2. Materials and Methods

**Figure 1:**
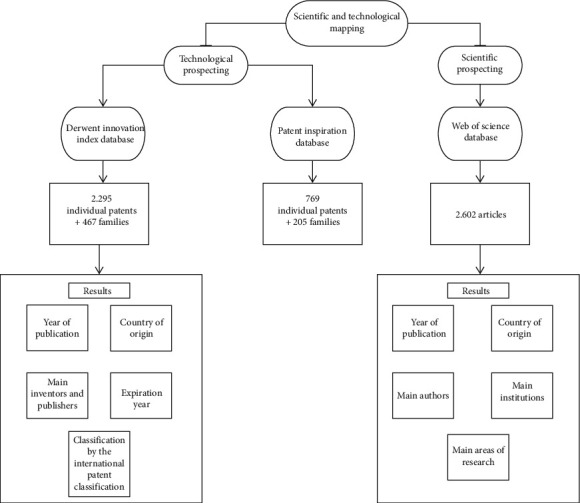
Flowchart of the methodology performed in this work.

The present study is exploratory research (Figure 1**)**, aiming to collect technical and qualified information with a patent and articles focus.

The technological prospecting was carried out in the Derwent Innovation Index® (Derwent World Patents Index-DWPI) database, Thomson Innovation©, with a license for use by University Center SENAI/CIMATEC. The databases must be evaluated according to some important criteria for a search focusing on patents. The search must be able to be comprehensive with a focus on the subject on which information is required, that is, the search must respond to the informational needs for that type of technology prospected. In addition, it should allow the retrieval of information with less complex search expressions and also be able to find relevant and current documents, from the speed in including new patent documents in the database [68]. Paid access tools, such as Derwent Innovations Index®, present as a differential; the speed in retrieving the information sought, being able to analyze a large number of data in a simplified and efficient way, allows the use of statistical tools and promotes the generation of information through graphics and maps, among others, allowing their analysis. The efficiency of this base can be directly related to the high cost of obtaining your license [56]. DWPI is a wide-ranging database represented by the US company Thomson Reuters, with indexed patents in the area of chemistry, engineering/electricity, and electronics with worldwide coverage, which contains more than 80 million practical inventions counted since 1983. With weekly update, only in 2019, six million patents were added. With several search options (general or advanced search) in several search fields, the recovery of documents can be carried out, besides the use of keywords and Boolean operators, from the International Patent Classification (IPC) and the classifications elaborated by the database itself [69].

In order to provide an overview of the amount of recovered patents compared to another database, PatentInspiration® was used in order to assess the difference in the number of records in each database. PatentInspiration is a database of the company AULIVE, which operates since the year 2000 in the field of information technology and services. It has a collection of practical and systematic tools, such as PatentInspiration, a platform based on the DOCDB database of the EPO (European Patent Office) that allows an analysis of more than 69 million patents in the areas of processes and products, from more than 90 countries. It has the proposal to support the innovative process, competitive intelligence, and obtaining technology maps. The indicators can also be found in this database through the search in several fields as well as the use of IPC classification codes [70, 71].

The data were obtained using a search strategy containing the IPC code C07K 16/00, related to immunoglobulins, such as monoclonal or polyclonal antibodies, since it helps to recover the patents with greater accuracy. In addition, keywords were used according to the search strategy:

(C07K 16/00) AND ((“monoclonal antibody”) AND (therapy OR “autoimmune disease”) NOT (methods OR device)).

In the DWPI database, associated with this strategy, the DWPI classification system was used, categorizing patent documents from a simple classification system for all technologies. It is an exclusive classification applied consistently to all patents by the specialists of Clarivate Analytics, allowing effective and precise research in the specific area of the methodology. Thus, code B04 was used, which is associated with “natural products and polymers, including testing body fluids (except blood group determination or cell count), veterinary or pharmaceutical compounds of an unknown structure, testing microorganisms for pathogenicity, testing chemicals for human mutagenicity or toxicity, and fermentative DNA or RNA production, and general compositions.” The use of this code will allow a comparative analysis between the numbers of records found in both databases.

The research was based on the search of the selected terms in the title, summary, and claims of the documents, in both databases. The period of data collection was based on the documents found in the last 28 years. The construction of the graphics was carried out with Origin 6.0 program from the results found in the DWPI database. It is worth mentioning that because of the secrecy period preceding the publication of the patent (18 months), some patents may not be shown in the research.

The indicators considered for analysis of the data were year of publication, the country of origin, the main inventors and publishers, the expiration year, and the classification of the documents by the International Patent Classification (IPC) established by the WIPO (World Intellectual Property Organization).

In order to broaden the discussion of this study, a bibliometric analysis focusing on the search for related scientific articles was conducted. In scientific terms, bibliometry is a tool that can be used for mapping scientific publications. From this analysis, it is possible to identify through the use of statistical analysis by the studies, the main topics related to mAbs research, international research trends, as well as the development status of these drugs [72, 73]. For this purpose, the Web of Science (WoS) database was used. WoS is a multidisciplinary scientific database of international scope that has a wide coverage of citation indexing, allowing the provision of the best data from scientific publications. The platform allows the tracking of ideas across disciplines and time from almost 1.9 million cited references from over 171 million records. In addition to the broad citation indexing coverage, WoS has valuable search functionality in the advanced search field, as well as allowing files to be saved in a variety of formats for data download [74].

The same search strategy was used in the prospection of scientific publications in WoS, without using the IPC code. The search was restricted to an analysis of articles published in the last 5 years, and the citation index used was Science Citation Index Expanded (SCI-EXPANDED), which is related to science area. The construction of the graphics was carried out with Origin 6.0 program from the results found at WoS for the following indicators: temporal evolution of the articles, main countries in terms of scientific publication, main authors of the publications found, main institutions, and main areas of research.

## 3. Results and Discussion

### 3.1. Patent Mapping

In the last 25 years, the use of mAbs has become predominant in the treatment of several diseases. Along this period, the importance of technological advances has proven to be effective in encouraging the discovery and development of faster and more efficient therapies. In 2019, the US-FDA approved 6 mAbs that are available on the market. They are caplacizumab (from Ablynx/Sanofi) indicated for thrombotic thrombocytopenic purpura, romosozumab (Amgen/UCB) for osteoporosis in women who are in the postmenopause period at an increased risk of fracture, risankizumab (Boehringer Ingelheim/AbbVie) used in treatment of plaque psoriasis, polatuzumab (Roche, F. Hoffmann-La Roche, Ltd.) indicated for diffusing large B cell lymphoma, and brolucizumab and crizanlizumab, both from Novartis, indicated for macular degeneration and sickle cell disease, respectively [75]. The approval of mAbs paves the way for related research, whether in the use of the drug for its proper purpose or in its reuse for a condition that does not fall within its scope of purpose. A new study that addresses the possible use of an FDA-approved monoclonal antibody called fostamatinib (from Rigel Pharmaceuticals, sold under the brands Tavalisse and Tavlesse) can be cited as an example. Fostamatinib is a spleen tyrosine kinase inhibitor (SYK) that acts in the reduction of mucin-1 levels, a molecule that is associated with cases of acute lung injury and acute respiratory distress syndrome (ARDS). In animal experimental models, this drug is acting efficiently in the treatment of severe inflammatory diseases (e.g., vasculitis and immune glomerulonephritis) [76]. In vitro and in vivo studies suggest that fostamatinib can be effective for the treatment of patients with severe conditions of COVID-19 (coronavirus disease 2019) [77, 78]. For 2020, the Bavencio vaccine (avelumab), used in the treatment of patients with cancer, was presented by the company Pfizer, and the company GlaxoSmithKline plc. has registered to introduce belantamab mafodotin, which will work on the maturation antigen of the B cells [79]. This and other studies are underway to “reuse” monoclonal antibodies in the treatment of other noncorrelated diseases.

Entering the discussion of the data generated in this study, after the technological prospecting was carried out, a total of 2295 individual patent documents and 467 families were found using DWPI database and 769 individual patents and 205 families using PatentInspiration. It should be noted that the patent family corresponds to a set of patent application deposits, in which all deposits have at least one priority document in common. They are published or granted in order to protect a single invention [80]. The technologies found were relative to technologies of biological products and/or mAbs. mAbs are produced by a single B cell clone and specifically target a specific antigen. Produced in the laboratory, these molecules act as substitute antibodies that can restore, improve, or imitate the “attack” of the immune system in the face of aggressions to the organism [81].

**Figure 2:**
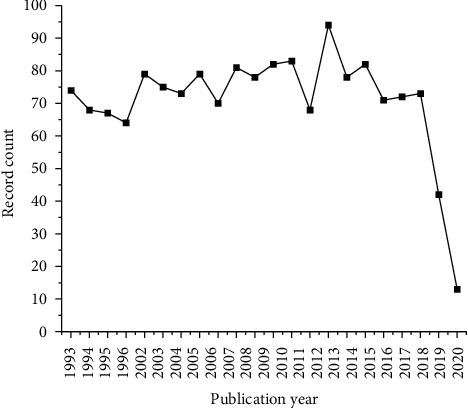
Patent publishing trends between years 1993 and 2020.

Figure 2 shows the trend in family patent publication from 1993 to 2020. Although the results do not show an increase in the number of publications each year, mAbs are still a class of drugs that are growing fast, pointing out 76 antibody-based therapies that have received approval for clinical use [82], probably identified within the prospective patent families. In 2019, 42 publications were identified, and there were 13 publications in 2020 (until the date of prospecting). It is worth mentioning, as explained previously in the methodology, that publications may have been left out of the results because of the secrecy period.

When comparing DWPI results with PatentInspiration results, only 205 families of patents were found in the last one. This smaller number may be associated with the fact that in addition to having used the free version of the database, the insertion of the DWPI's proper classification code may have helped in a greater recovery of patent documents. As for the publication trend, in PatentInspiration, the year 2020 was the most remarkable, with 19 patents published.

**Figure 3:**
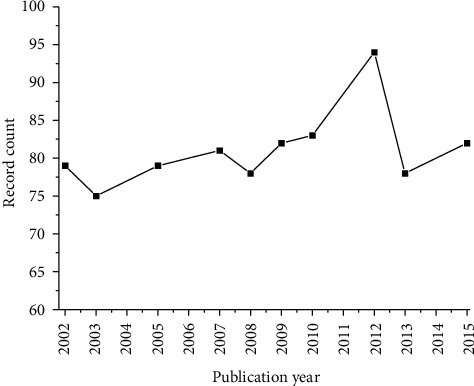
Top 10 years related to the number of published records.

Figure 3 shows the top 10 years that had more publications in the evaluated period. The year 2012 is in first place, with a total of 94 publications, followed by 2010, with 83 publications. These data corroborate with the almost tripled increase in the amount of therapeutic mAbs on the market between 2010 and 2019 [83].

The trend of continuous growth of mAbs as an effective therapy in the treatment of diseases, especially cancer and autoimmune diseases, is based on the high power and specificity of the binding between antibodies and specific targets, together with the ability to access a wide variety of mechanisms of action, resulting in good tolerance of the organism and less risk for unforeseen problems, as in many other treatments [84]. With regard to protected patents, published between the years 2019 and 2020, there were 13 publications in 2020 and 42 in 2019. Some of them are described in Table 1.

**Figure 4:**
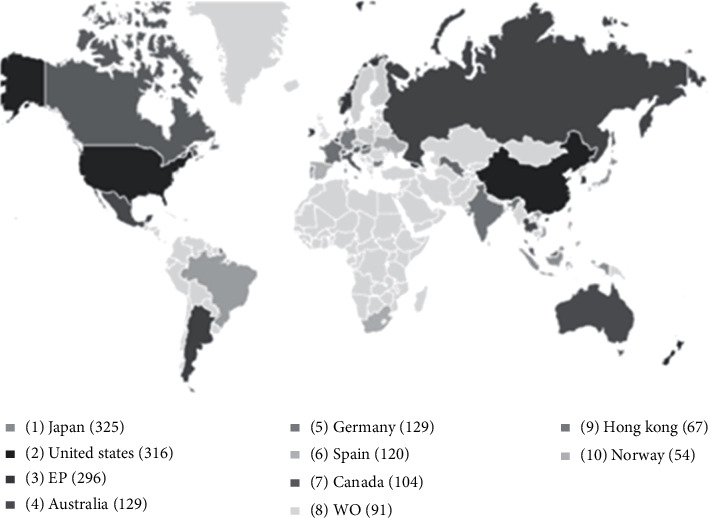
Top 10 countries/organizations that most contribute in terms of number of publications (obtained by DWPI database).

When analyzing the countries that contribute the most in respect of the quantity of publications in prospective patent families, it is possible (Figure 4) that Japan and the USA occupy the first and second place, respectively. The results showed that developed countries are responsible for the greatest interest and domain in the technology investigated in this study.

Unlike the result found in the DWPI, in the prospection carried out in the PatentInspiration database, the USA appears in first place at the top of the main countries that most contribute to the publication of patents in the area of mAbs, with 47 publications. Japan appears in second with 42 and Germany in third with 24 publications.

Within the scope of mAb technologies, current research is mostly concentrated in developed countries, as shown in Figure 4. In many of these countries, the clinical development of monoclonal antibodies began with the production of murine mAbs [93]. Although patents are one of the least publicized indicators, they are key elements to measure the advancement of technologies in countries and, perhaps more importantly, the impact they have on the country's technological development, as they represent the real creation and dissemination of knowledge in the areas that are productive. In addition to ensuring monetary benefits through marketing, companies still gain from the sale or licensing of technology, favoring the dynamism of knowledge and technologies that positively have an impact on society [94].

The use of patents as a defensive instrument is also widely used as an anticompetitive practice by large countries to avoid strategies such as blocking techniques (acquisition and not using new patents) and fencing (patent application for any possible technology that can be used) the entry of new competitors in the market, as well as advancing in relation to direct competitors [95]. This fact directly influences the market value of these drugs. In 2019, the global mAbs market was sized at USD 37.13 billion, and studies estimate that growth for the coming years will be at a rate of 5.30% CAGR (compound annual growth rate) reaching a value of USD 63.13 billion in 2024. Increasing incidence of important diseases such as diabetes and cancer is without a doubt another key point that explains the growth of the global mAbs market. Moreover, the high investment in the area and the high demand to ensure quality in health service allows the invention of new applications of mAbs, which also explains the accelerated need of the market and significantly influencing the fulfillment of world demand [79].

In terms of market structure, most pharmaceutical product sales (in general) are from developed countries. This is because most pharmaceutical companies operate with a number of expertise and practices that involve researchers in a multidisciplinary way. In addition, thanks to the great support of private companies for the public sector, in developed countries, technoscientific production stands out [96].

**Figure 5:**
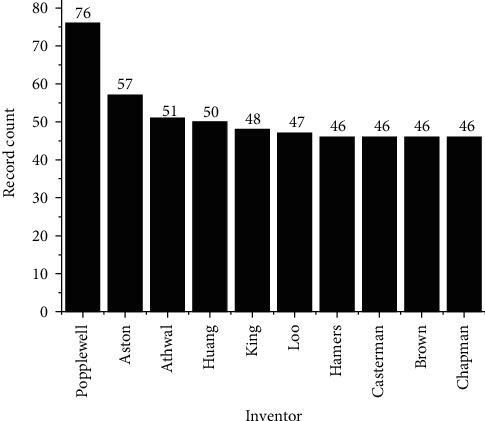
Main inventors related to the number of publications.

Among the main inventors highlighted in the technological prospecting of patents associated with mAbs, we highlight Andrew G. Popplewell, Roger Aston, Singh D. Athwal, Ling Huang, David John King, Deryk Loo, Raymond Hamers, Cecile Casterman, Thomas D. Brown, and Paul A. Chapman, as shown in Figure 5.

All of these authors have published patents in association with universities or by pharmaceutical companies in the field of biological production. When the comparison between the databases was made, in the search for the identification of inventors, none of the main ones highlighted in the DWPI search was identified in the PatentInspiration. This can be explained by the difference in document coverage between the databases, with respect to the scope of patent filing offices, which makes the results found between the databases distinct.

**Figure 6:**
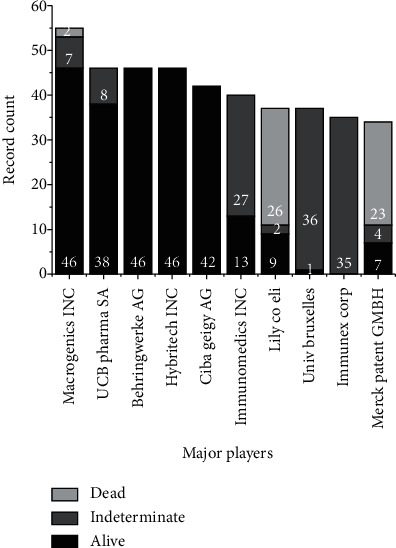
Major players related to the number of publications.

As shown in Figure 6, which identifies the biggest competitors in the pharmaceutical industry, the company MacroGenics Inc. has 20% (9 records) more than its closest competitor, UCB Pharma SA.

MacroGenics Inc. is headquartered in Rockville, Maryland, USA, and UCB Pharma SA is in Brussels, Belgium. As a biopharmaceutical company, MacroGenics Inc. is focused on discovering and developing innovative antibody-based therapies, designed to modulate the human immune response to cancer treatment, whose scientific experience is in the field of protein engineering through proprietary platforms such as DART® bispecific and Fc Optimization platforms, generally focused on the creation of antibodies and derivatives and antibody-like molecules [97].

Compared to the other 10 largest players, MacroGenics Inc. owns 13% of document records, with 46 active records and 7 shown as indeterminate. Among the companies observed, the University of Brussels stands out with a total number of 37 records, of which 2 are active and 35 are indeterminate.

**Figure 7:**
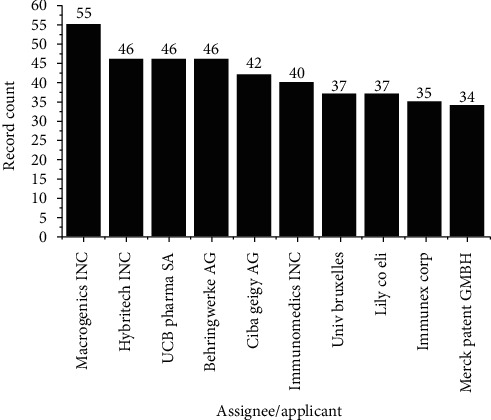
Top 10 assignees/applicants related to the number of record counts.

Regarding the 10 largest assignees/applicants, MacroGenics Inc. remains in the first position as shown in Figure 7 and in second place, Hybritech Inc., the San Diego-based subsidiary of Eli Lilly and Co., with 55 and 46 record counts, respectively.

In comparison with PatentInspiration, differences were also found in the search for the main applicants of the patent documents. Only Ciba Geigy AG, Immunomedics Inc., and Merck Patent GMBH were identified in the prospection in this database, but not among the 10 main applicants. Moreover, only 3 records were identified for Ciba Geigy, 2 records for Immunomedics Inc., and 3 for Merck Patent GMBH, being these results different from those found in the DWPI database.

**Figure 8:**
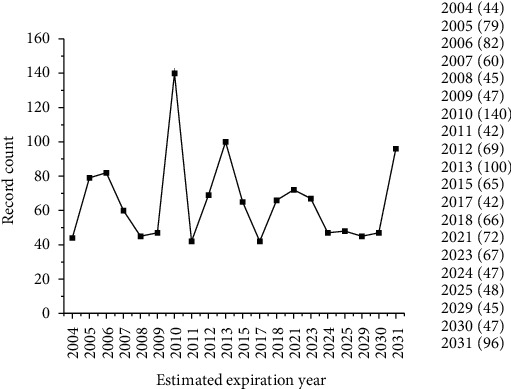
Estimated expiration year of the publications.

In terms of time, Figure 8 shows the year of expiration of the prospected documents, indicating that it is estimated that 422 documents in the area of biological products or related to monoclonal antibodies will expire in 2021. This analysis is important because the expiry of patents on reference biologics is an essential factor in reducing the costs of production and marketing of biosimilars.

It is important to note that drug prices decrease significantly after patent expiry, and this price reduction varies between products and countries. Therefore, country-specific analyses on the evolution of prices after the expiry of the patent are usually performed. These analyses are important for decision making regarding the research and production of these drugs [98]. Because they are versions of their reference biologicals, the production of biosimilars is usually cheaper because of the abbreviation in the clinical trial program, which directly impacts the achievement of a more advanced and efficient process. By 2022, an increase in wholesale prices of 8%–11% is expected, while for net prices, 2%–5%, per year. In the last 10 years, there has been an increase of up to tenfold in the drugs against cancer. Now, the median annual return is at least US$150.000 [99]. By 2021, as shown in the results in Figure 8, 422 documents are expected to have their patents expire. This means that biologics that are currently marketed may suffer discounts and value reductions because of the introduction of biosimilars in the market in the future. A British study has evaluated the savings generated by the use of the etanercept biosimilar GP2015 in the treatment of patients with rheumatoid arthritis or psoriasis. The results showed that GP2015 can offer substantial cost savings. In a 10% discount scenario, the economy could reach GBP 4.8 million, while in a 30% discount scenario, GBP 14.3 million. The authors determined that this reduction would allow for an additional 568 patients (10%) and 2191 patients (30%) to be treated with this drug [100]. Based on the budget model, another study identified that the use of the biosimilar rituximab CT-P10 could generate savings of 90 million euros in Europe in the first year alone. The same would not be observed in relation to the use of its biological reference [101].

The biosimilars, in general, consist of an opportunity to stimulate competition and lower treatment costs, without compromising the clinical results of the studies. In order to contribute to the broad use of biosimilars, in addition to the financial benefits already discussed, the education of clinicians and patients on the safety and equivalence of drugs to be used and the effect of their use in healthcare is also an important strategic approach to promote the dissemination of biosimilars. This strategy based on clinical guidelines may be fundamental, but despite this, payment programs and value-based care models must be taken into consideration, since they can act as major influencers related to the greater financial risk for healthcare administered by doctors and the treatment received by patients [102]. In every biosimilar production process, quality is always a parameter that is extensively evaluated and compared to the respective biological reference. The pharmacokinetic profile of monoclonal antibodies is significantly affected by the related structures (e.g., glycan structure and isoelectric point), in addition to the ability of the antibody to bind to the target antigen. For this reason, in order to validate a biosimilar, its profile pharmacokinetics must be comparable to the reference biological, and this comparison is made from clinical and nonclinical studies, in which the results obtained must demonstrate high similarity in terms of the necessary quality attributes [103].

These data corroborate the significant increase expected in the branch of biological production, mainly the production of biosimilars, since upon document expiration and entry of the technology into the public domain, other companies can take advantage and act in the branch of innovation with the introduction into the market of similar and lower-cost products, paving the way for pharmaceutical companies in the industry to prosper [18, 21].

**Figure 9:**
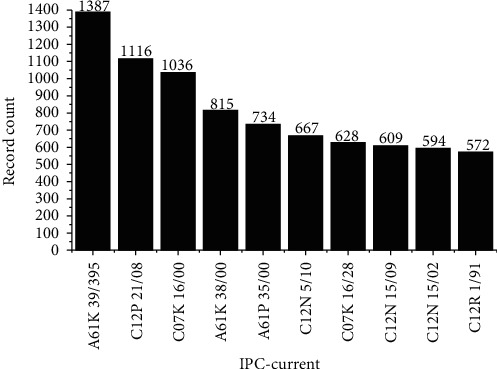
Current IPC codes used in the classification of the prospected patents.

As a last result, the list of IPC codes used for the classification of patents is shown in Figure 9. The code A61K 39/395 was the most commonly used, for 1387 records, followed by the code C12P 21/08, for 1116 records. All patents were classified in the broad areas of “Human Necessities (section A)” or “Chemistry; Metallurgy (section C).” Table 2 shows the codes mentioned above, in addition to the others found.

Patent prospection is important for the evaluation of the innovation process. The research and patenting of technologies involving mAbs allow greater movement in the market of monoclonal antibodies, as well as encouraging scientific research in view of its possibilities for use. Technologies include those identified in patents JP06416926 B2, which refers to the use of tumor targeted interleukin-12 (IL-12) at the induction and/or stimulation of the immune response against cancer [104]; ES2687706 T3, which refers to the use of monoclonal antibodies or immunoreactive fragments that react with the region of the hemagglutinin protein stalk of viral clades of influenza, used to treat infection by the influenza virus [105]; and EP3059314 B1, which addresses the development of a new antibody specific to the human tumor necrosis factor- (TNF-) alpha, useful in the treatment of diseases such as congestive heart failure, cachexia, endotoxic shock, and adult respiratory distress syndrome, all mediated by TNF-alpha [106]; among many other studies found in this prospecting, these patents substantiate the importance of research related to monoclonal antibodies, since their use may cover many more medical conditions than just the treatment of cancer and autoimmune inflammatory diseases.

### 3.2. Scientific Mapping

It is important to say that there is a relationship between patents and articles. Many corporations have been reducing research production and focusing on development over the years, not because research is less relevant to innovation but because many companies are not willing to invest time in the maze of science, preferring to rely on or invest in universities, national laboratories, and other research institutes [107]. This relationship encourages an increase in the number of studies that can be used as reference sources for the development of protectable technologies, as well as allowing the possibility of investigating the use of medicines already available on the market for their application in the treatment of other medical conditions.

**Figure 10:**
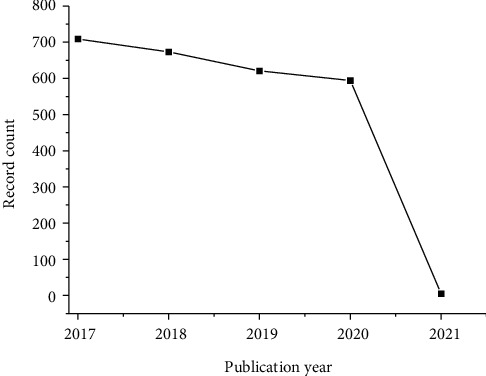
Temporal evolution of articles published in the last 5 years.

After the scientific prospecting was carried out, 2602 articles were found using WoS database.  Figure 10 shows the temporal evolution of scientific articles published in the last 5 years. The year 2017 counts with the largest number of publications in relation to prospected technology with a total of 709 articles. After this year, a reduction in the number of publications was observed, being 673, 621, and 594 publications in the years 2018, 2019, and 2020, respectively. The analysis showed that in 2021, the publications of 5 articles have already been published/are expected, being 2 of them related to the area of pharmacology and the others in the areas of molecular biology/biochemistry (1), computer science (1), crystallography (1) endocrinology/metabolism (1), mathematical/computational biology (1), and oncology (1).

In the last 25 years, mAbs have become a predominant modality in the treatment of several diseases. Throughout these years, the focus of scientific research was based on making faster the discovery, as well as the development of therapies aimed at the use of mAbs [75], and although a reduction in the number of publications has been observed, this does not indeterminate the research in this area. This can be explained by the fact that research on mAbs has become more and more interdisciplinary, making it more difficult to obtain an understanding of all development related to mAbs [72].

**Figure 11:**
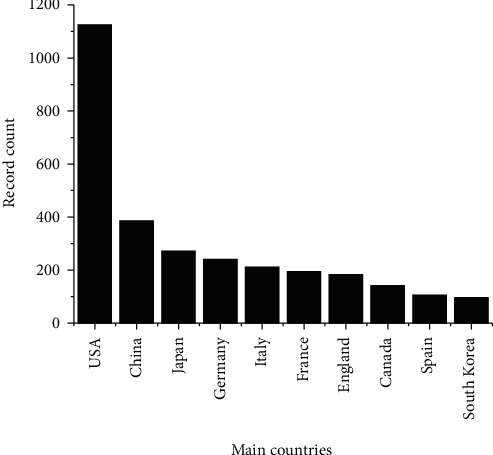
Top 10 countries that most contribute in terms of number of publications.

Notwithstanding the number of publications, when the analysis of the main contributing countries in publications in this area was performed, it is shown in Figure 11 that the USA represents approximately 43.2% of the scientific articles recovered, totaling 1125 articles. In second place was China, with 385 publications, followed by Japan (272), Germany (241), Italy (211), France (194), England (183), Canada (141), Spain (106), and South Korea (96). Altogether, more than 80 countries/regions were mapped across the continents. Some countries highlighted in scientific publications also stood out in patent publications, such as Japan and the USA.

**Figure 12:**
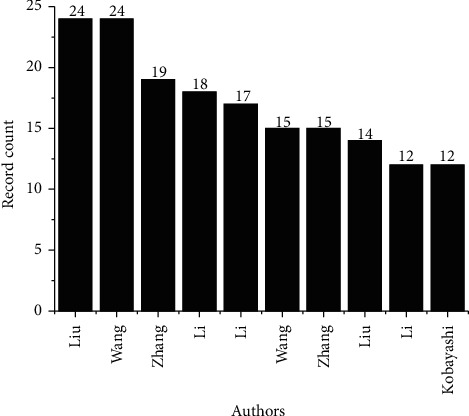
Main authors of published articles.

The top 10 authors among the more than 18 thousand identified in the publications that integrate the database are presented in Figure 12. Among the most productive researchers are Liu and Wang responsible for 24 articles each, followed by Zhang with 19, Li with 18, Li with 17, Wang and Zhang with 15, Liu with 14, and Li and Kobayaski with 12 articles each. The most prominent area among these authors is oncology, being characteristic of 7 of these main cited authors. Other corresponding areas are science technology, research experimental medicine, and material science. In order to evaluate the works of these main authors, the most recent articles published by them were analyzed according to their predominant areas. Among the 3 main authors, Liu participated in a study that evaluated the efficacy of an anti-CD137 antibody, with the objective of triggering anticancer responses. CD137 is a costimulating molecule that acts on the activation of T cells, regulating the activity of these cells in physiological and pathological processes [108]. Wang was one of the authors of a study that tested two types of mAbs for the treatment of hemophilia [109], and Zhang participated in a study whose objective was to evaluate the performance of monoclonal antibody 7C6 in marking MICA/B a3, a domain present in tumor cells, potentiating the activity of antitumor CIK cells [110]. Other studies of the main authors cited and other researchers are given in Table 3.

**Figure 13:**
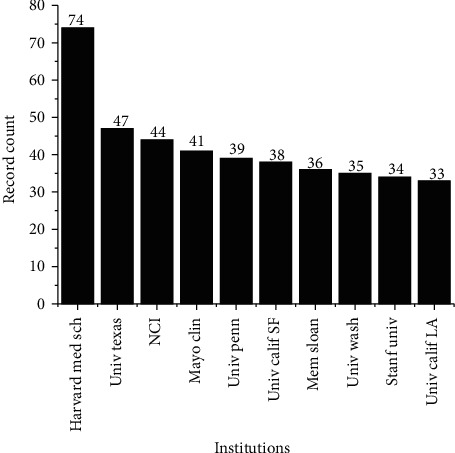
Main institutions responsible for scientific publications.

Figure 13 shows the top 10 organizations that have published the most articles related to biological products and/or mAbs from a total of more than 4 thousand identified. Harvard Medical School stands out with 74 records, followed by the University of Texas MD Anderson Cancer Center with 47, National Cancer Institute (NCI) with 44, Mayo Clinic with 41, University of Pennsylvania with 39, University of California in San Francisco with 38, Memorial Sloan-Kettering Cancer Center with 36, University of Washington with 35, Stanford University with 34, and University of California in Los Angeles with 33. The importance of research in universities and research centers is of great relevance, mainly due to the fact that a decline in corporate research (companies/industry) has been observed, which has been investing mainly in innovation and development of technologies and products [107]. The great prominence of these organizations present mainly in developed countries can be given to the fact that, in these countries, there is a greater investment in research and development triggered by universities and research centers.

**Figure 14:**
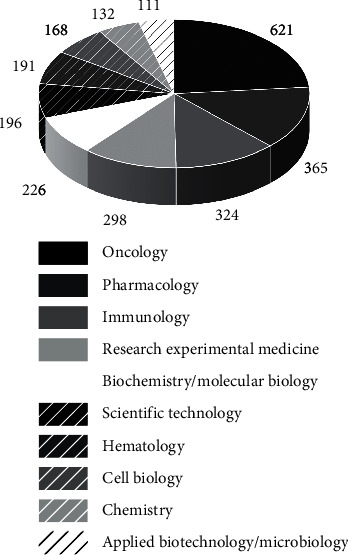
Main research areas of the scientific articles found.

As a last analysis, the main research areas related to mAbs were identified. In all, 81 research areas were identified, where the 10 main ones are highlighted in Figure 14. The sum of the number of articles identified in all areas is greater than the number of prospect records in the database; however, this can easily be related to the fact that a single article may be associated with more than one area of knowledge. The oncology area was the most prominent area in the search, accounting 621 published articles. In second place is the area of pharmacology with 365 records, followed by immunology with 324, research experimental medicine with 298, biochemistry/molecular biology with 226, science technology with 196, hematology with 191, cell biology with 168, chemistry with 132, and biotechnology applied/microbiology with 111 records of scientific publications. The fact that oncology is the most predominant area of knowledge may indicate the greater direction of mAbs research for the treatment of cancer, and this type of research is being considered extremely valuable and, according to the data found in the articles surveyed, very successful [81]. In general, as far as research lines with mAbs are concerned, most studies are representative of the preclinical phase and/or in the discovery phase. It is worth considering that some of these studies may currently be part of discontinued research programs.

It is important to highlight that the growth in the oncology area does not mean that there are no other directions for the research and application of mAbs. The investigation for inflammatory and chronic conditions has also been showing great efficacy in the results obtained in several research studies described. Speculating for the future, it is possible to say that several other directions can be given to molecular biology based on the production of antibodies, thus offering greater therapeutic opportunities.

The growth in the use of mAbs in therapeutic treatments was considerably leveraged by the increase in the knowledge capacity acquired in relation to the molecular basis of diseases, as well as the discovery of modern technologies such as phage display [118, 119]. The scenario evaluated in this study pointing to the importance in the number of scientific and technological publications may help to confirm the growth in this area of research. The scientific mapping and patent are of utmost importance to contribute with analyses regarding the growth of research and technological development in mAbs, allowing a connection to be made between the growth trend in the number of articles and patents as a signal for future investments in mAbs. The development of a strong literary base on mAbs allows the identification of new research in development, thus allowing the measurement and quantitative analysis regarding all parameters about this technology and investment possibilities [73]. According to some authors, the information found in patent documents is considered as one of the best sources for identifying technological changes in the sector to be studied [56]. However, it is worth mentioning that the knowledge acquired through patent documents and the technological advances provided are directly related to advances in basic research [81], usually reported in scientific articles.

It is possible to affirm that, with the progress of technologies in the area and the emerging expansion of the market under mAbs technologies, there is a positive trend of research and development that promises to diversify the use of this therapeutic form bringing opportunities for new clinical applications. The growing impulse of research activities and the accessibility of technology to developing mAbs also contribute to market growth and financial return [120], which may be associated with the growing prevalence of biosimilar mAbs. The biosimilars result in lower costs of both production and treatment, costing about 20–25% less than the reference biological. In addition, as a way to strengthen the market, the main companies bet on collaborations and acquisitions of partnerships, as well as the purchase of startups to expand their portfolios of services and products [121]. It is worth mentioning that other opportunities can be identified with the advance of mAbs research. The growing awareness of therapeutic applications of mAbs to patients, the identification of better products, and the insertion of these antibodies in markets can enhance their use in cost sensitive markets which, based on available scientific and technological information, can also contribute to the agility of the approval processes of these therapies by regulatory agencies.

## 4. Conclusions

Technology research through the knowledge made available in scientific and technological publications can widely contribute to decision making by companies, as well as direct scientific and clinical research in mAbs. The search through database constitutes a systematic tool for mapping the current situation of technologies, and as seen in the results of this study, depending on the type of database used, different results can be found. For this reason, the choice of the database to be used is of extreme importance for the projection of technological and scientific trends. According to the results found, advances in targeted therapies using monoclonal antibodies have generated intense interest for decades. These drugs have become essential weapons in the treatment of significant diseases, such as cancer and autoimmune diseases, and the trend in advancing the number of related patents and scientific articles contributes to making these therapies available to the greatest number of people. Based on the number of publications registered, it is expected that several therapeutic drugs are already under regulatory review, enabling approvals for the next few years, thus generating a continuous flow of new products. In this sense, therefore, it is believed that the arrival of these drugs may contribute to the prevention and treatment of diseases, based on the use of therapy based on monoclonal antibodies. In addition, along with the contribution promoted by scientific research reported in articles, access to new drug trends as well as possibilities of use help to expand the range of options for the use of mAbs in a variety of disease treatments.

## Figures and Tables

**Table 1:** Description of some patents published between 2019 and 2020.

Publication number	Title	Publishing date	Description
CN106103466 B	New blood-brain barrier shuttle comprising a brain effector entity and a brain targeting peptide, useful for treating Alzheimer's disease.	March 31, 2020	The invention provides a blood-brain barrier shuttle comprising a brain effector entity and a target brain peptide [85].
IN334250 B	New drug conjugate comprising drug moiety covalently attached to rest of drug conjugate is human epidermal growth factor receptor 2 inhibitor, useful for treating cancer, preferably lung, colorectal, breast, and pancreatic cancers.	March 13, 2020	New drug conjugates, drug-binding compounds, pharmaceutical compositions containing said drug conjugates and their use as antitumor agents. The researchers took advantage of the cell surface receptors and antigens selectively expressed by target cells, such as cancer cells, to develop pharmaceutical entities based on antibodies that bind, in the example of tumors, specific or tumor-associated antigens [86].
ES2742351T3	New isolated antibody or its immunoreactive fragment comprises a variable domain that specifically binds an extracellular domain of B7-H3, useful for diagnosing cancer, and for preparing a medicament for treating cancer in a patient.	February 14, 2020	Antibodies and their fragments that are immunoreactive against the mammalian, and more particularly human, B7-H3 receptor and uses thereof, particularly in the treatment of cancer and inflammation. The invention therefore relates particularly to humanized B7-H3 reactive antibodies and their immunoreactive fragments that can mediate and more preferably enhance activation of the immune system against cancer cells that are associated with a variety of human cancers [87].
JP06642946 B2	New ligand drug conjugate compound comprising beta-glucuronide based linker for treating cancer, autoimmune disease, and infectious disease.	February 12, 2020	A ligand drug conjugate comprising a ligand, such as an antibody, provided for allowing the conjugate to target a target cell or target tissue. The conjugate further comprises a *β*-glucuronide-based linker including a site that can be cleaved by an enzyme having *β*-glucuronidase activity. The linker is bound to the ligand and the drug. According to the present invention, furthermore, there is provided a method for treating cancers, immune diseases, infectious diseases, and other diseases or disorders using the ligand drug conjugate comprising the *β*-glucuronide-based linker [88].
AU2020200175 A1	New antibody-drug conjugate compound where a linker drug is site-specifically conjugated to an antibody through an engineered cysteine for use in treating solid tumors and hematological malignancies, e.g., breast cancer and prostate cancer.	January 30, 2020	Relates to antibody-drug conjugates (ADCs), wherein a linker drug is site-specifically conjugated to an antibody through an engineered cysteine, and their use as a medicament, notably for the treatment of human solid tumors and hematological malignancies, in particular breast cancer, gastric cancer, colorectal cancer, urothelial cancer, ovarian cancer, uterine cancer, lung cancer, mesothelioma, liver cancer, pancreatic cancer, prostate cancer, and leukemia [89].
CA2665480C	New peptide conjugate comprising a peptide which is covalently attached to a moiety, useful for preparing a composition for treating tumor.	November 11, 2019	Production of conjugates between peptides and PEG moieties through glycerol linkers. In one aspect, the present invention provides a conjugate between a PEG moiety, e.g., PEG and a peptide that has an in vivo activity similar or otherwise analogous to art-recognized therapeutic peptide. In the conjugate of the invention, the PEG moiety is covalently attached to the peptide via an intact glycosyl linking group. Exemplary intact glycosyl linking groups include sialic acid moieties that are derivatized with PEG [90].
BRPI0608281 B1	Human monoclonal antibody or its fragment, binding to and neutralizing primate granulocyte-macrophage colony stimulating factor, useful for treating inflammatory diseases and cancer.	January 28, 2020	A human monoclonal antibody or fragment of its own which specifically binds GM-CSF and neutralizes it [91].
NO344249 B1	Human monoclonal antibody or its fragment, binding to and neutralizing primate granulocyte-macrophage colony stimulating factor, useful for treating inflammatory diseases and cancer.	October 21, 2019	A human monoclonal antibody or fragment that specifically binds to and neutralizes primate GM-CSF [92].

**Table 2:** International Patent Classification (IPC) established by the WIPO, found in prospective patent documents.

IPC code	Code description
A61K 39/395	Antibodies (agglutinins A61K 38/36); immunoglobulins; immune serum, e.g., antilymphocyte serum.
A61K 38/00	Medicinal preparations containing peptides.
A61P 35/00	Antineoplastic agents.
C07K 16/00	Immunoglobulins, e.g., monoclonal or polyclonal antibodies.
C07K 16/28	Against receptors, cell surface antigens, or cell surface determinants.
C12N 5/10	Cells modified by introduction of foreign genetic material, e.g., virus-transformed cells.
C12N 15/09	Recombinant DNA technology.
C12N 15/02	Preparation of hybrid cells by fusion of two or more cells, e.g., protoplast fusion.
C12P 21/08	Monoclonal antibodies.
C12R 1/91	Cell lines.

**Table 3:** Description of some recent studies on mAbs prospected in WoS database.

Title	Journal	Publication year	Description
Antitumor effect of humanized antitissue factor antibody-drug conjugate in a model of peritoneal disseminated pancreatic cancer	Oncology Reports	2020	The work evaluated the action of an original humanized anti-TF monoclonal antibody conjugated with monomethyl auristatin E against the tissue factor (TF), an attractive target involved in the overexpression of a wide variety of malignant cancers, through in vivo efficacy trials [111]
Acquired resistance to HER2-targeted therapies creates vulnerability to ATP synthase inhibition	Cancer Research	2020	The work evaluates the effectiveness of the combination of ATP oligomycin A inhibitor with trastuzumab oligomycin A in order to lead to the regression of tumors resistant to this antibody, such as HER2+ breast cancer patients [112]
InsB9-23 gene transfer to hepatocyte-based combined therapy abrogates recurrence of type 1 diabetes after islet transplantation	Diabetes	2021	The work evaluates a treatment option for autoimmune diabetes by using a lentiviral vector that allows the expression of the insulin B chain 9–23 in combination with a suboptimal dose of anti-CD3 monoclonal antibody [113]
Efficacy and safety of bevacizumab in advanced lung adenocarcinoma patients with stable disease after two cycles of first-line chemotherapy: A multicenter prospective cohort study	Thoracic Cancer	2020	This work is based on a clinical trial that aimed to evaluate the effects after treatment of patients with advanced pulmonary adenocarcinoma after two cycles of platinum-based double agent chemotherapy compared to patients with the same disease and same treatment but in association with bevacizumab [114]
Computational engineering the binding affinity of adalimumab monoclonal antibody for designing potential biosimilar candidate	Journal of Molecular Graphics and Modeling	2021	The work addresses a modeling study using bioinformatics tools to find variants of the adalimumab monoclonal antibody with improved binding properties, since some risk factors are associated with high dosages of this antibody in treatments of diseases such as rheumatoid arthritis and other autoimmune diseases [39]
Development of a dinutuximab delivery system using silk foams for GD2 targeted neuroblastoma cell death	Journal of Biomedical Materials Research Part A	2020	The work evaluates the action of dinutuximab as a treatment for patients with high-risk neuroblastoma, from a delivery system capable of delivering this bioactive antibody locally, using silk fibroin. This delivery system can potentially reduce the probability of cancer recurrence, as well as minimize side effects [115]
Treatment with tocilizumab for patients with COVID-19 infections: A case-series study	Journal of Clinical Pharmacology	2020	The work is based on an evaluation of the clinical impacts of the use of tocilizumab in the treatment of COVID-19 patients, based on a comparison between patients who received monoclonal antibody as treatment and patients who received other therapeutic alternatives [116]
Anti-complement C5 therapy with eculizumab in three cases of critical COVID-19	Clinical Immunology	2020	The work evaluated an alternative for the treatment of critical patients at COVID-19. The treatment is based on the use of a monoclonal antibody anticomplement, the anti-C5 antibody eculizumab, in the reduction of severe clinical manifestations such as respiratory failure and acute kidney damage [117]

## Data Availability

All results obtained with this scientific and technological prospection are included within the article.
